# Inferring the evolutionary histories of divergences in *Hylobates* and *Nomascus* gibbons through multilocus sequence data

**DOI:** 10.1186/1471-2148-13-82

**Published:** 2013-04-12

**Authors:** Yi-Chiao Chan, Christian Roos, Miho Inoue-Murayama, Eiji Inoue, Chih-Chin Shih, Kurtis Jai-Chyi Pei, Linda Vigilant

**Affiliations:** 1 Department of Primatology, Max-Planck Institute for Evolutionary Anthropology, Deutscher Platz 6, Leipzig, 04103, Germany; 2 Gene Bank of Primates and Primate Genetics Laboratory, German Primate Center, Kellnerweg 4, Göttingen, 37077, Germany; 3 Wildlife Research Center, Kyoto University, 2-24 Tanaka-Sekiden-cho, Sakyo-ku, Kyoto, 606-820, Japan; 4 Graduate School of Science, Kyoto University, Kitashirakawa Oiwake-cho, Sakyo-ku, Kyoto, 606-8502, Japan; 5 Animal Division, Taipei Zoo, No.30 Sec.2 Xinguang Rd, Taipei City, 11656, Taiwan; 6 Institute of Wildlife Conservation, National Pingtung University of Science and Technology, No.1, Xuefu Rd, Neipu Township, Pingtung County, 91201, Taiwan

**Keywords:** Species tree, Isolation with migration, Gene flow, Autosomal loci, Phylogenetic relationships, *Hylobates*, *Nomascus*, Divergence process

## Abstract

**Background:**

Gibbons (Hylobatidae) are the most diverse group of living apes. They exist as geographically-contiguous species which diverged more rapidly than did their close relatives, the great apes (Hominidae). Of the four extant gibbon genera, the evolutionary histories of two polyspecific genera, *Hylobates* and *Nomascus*, have been the particular focus of research but the DNA sequence data used was largely derived from the maternally inherited mitochondrial DNA (mtDNA) locus.

**Results:**

To investigate the evolutionary relationships and divergence processes of gibbon species, particularly those of the *Hylobates* genus, we produced and analyzed a total of 11.5 kb DNA of sequence at 14 biparentally inherited autosomal loci. We find that on average gibbon genera have a high average sequence diversity but a lower degree of genetic differentiation as compared to great ape genera. Our multilocus species tree features *H. pileatus* in a basal position and a grouping of the four Sundaic island species (*H. agilis*, *H. klossii*, *H. moloch* and *H. muelleri*). We conducted pairwise comparisons based on an isolation-with-migration (IM) model and detect signals of asymmetric gene flow between *H. lar* and *H. moloch*, between *H. agilis* and *H. muelleri*, and between *N. leucogenys* and *N. siki*.

**Conclusions:**

Our multilocus analyses provide inferences of gibbon evolutionary histories complementary to those based on single gene data. The results of IM analyses suggest that the divergence processes of gibbons may be accompanied by gene flow. Future studies using analyses of multi-population model with samples of known provenance for *Hylobates* and *Nomascus* species would expand the understanding of histories of gene flow during divergences for these two gibbon genera.

## Background

**Figure 1:**
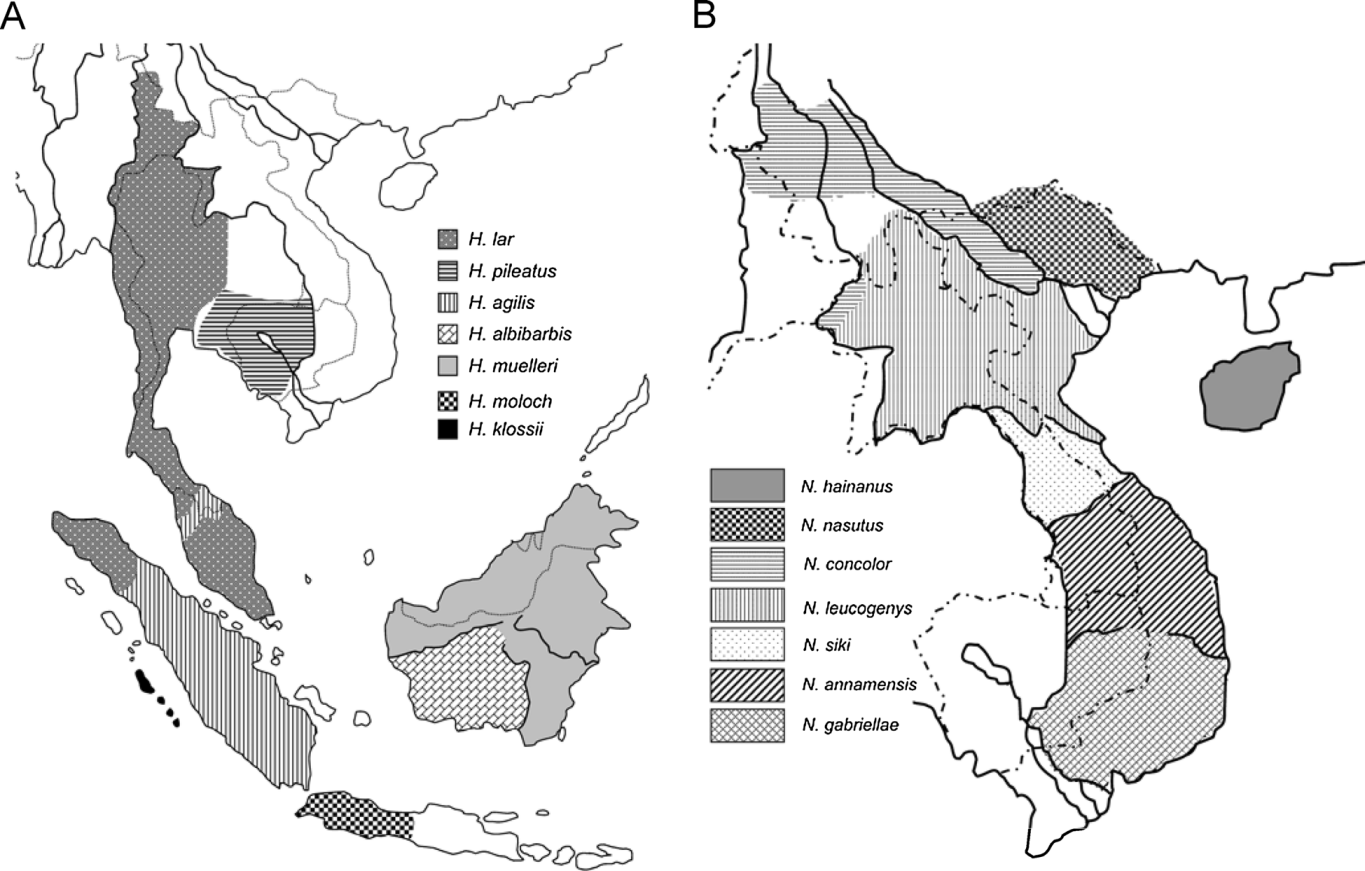
**Approximate geographic distribution of *Hylobates *(A) and *Nomascus *(B) species.** Dotted and solid lines indicate country borders and major rivers, respectively. Adapted from [10,12].

Gibbons are a family (Hylobatidae) of ape species endemic to the rainforests of the mainland and islands of Southeast Asia, including the Malay Peninsula, Sumatra, Borneo, Java and Mentawai Islands (Figure 1). They are the closest relatives of the great ape family (Hominidae) to which humans belong, and offer an interesting opportunity for comparisons. Like present-day great apes, the extant gibbons comprise four genera, which feature strikingly different numbers of chromosomes and extensive rearrangement of chromosomes [1-6]. Some 14 to 19 gibbon species have been recognized and classified into the genera *Hylobates*, *Hoolock*, *Nomascus*, and *Symphalangus*, indicating that gibbons consist of many more species than the seven species comprising the current day great apes [1,7-13]. In contrast to the geographically-discontinuous distribution of current-day great apes, gibbons now or recently live in close geographic proximity to one another. Moreover, the divergence of the four extant great ape genera (*Homo*, *Gorilla*, *Pan* and *Pongo*) apparently occurred over a time span of more than five million years [14-16], whereas the radiation of the four gibbon genera may have occurred within less than two million years [10,17]. Due to their close relationship to great apes, high taxonomic diversity and rapid diversification, gibbons have increasingly been the subject of molecular genetic analyses (e.g. [10,17-23]).

**Figure 2:**
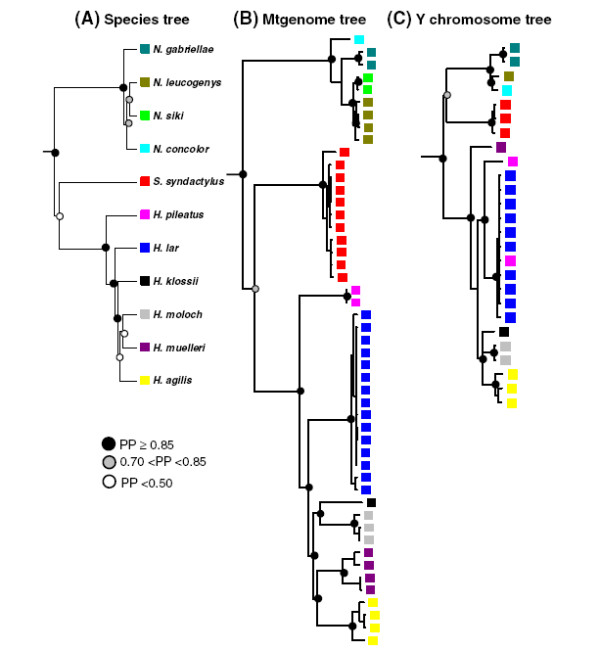
**Bayesian phylogenetic trees of gibbons.** (**A**) Species tree inferred based on sequences from 14 autosomal loci of 44 gibbon individuals (this study). (**B**) Mtgenome tree inferred based on mtgenome sequences of 49 gibbon individuals excluding the control regions (Additional file 1; [18]). (**C**) Y chromosome tree inferred based on the partitioned concatenated dataset of seven Y chromosomal regions of 26 gibbon individuals [19]. The support values for the nodes according to the Bayesian posterior probability (PP) are shown as the circles (filled circles: PP ≥0.85; grey circles; 0.70 < PP < 0.85; open circles: PP <0.50). The colored boxes indicate species of taxa.

Analyses of chromosomal number and structure and sequences of mitochondrial DNA (mtDNA) and nuclear loci consistently find that all four gibbon genera are monophyletic [3,10,17-21,24-26], although the exact phylogenetic relationships among the genera are still debated. Different phylogenetic trees have been proposed, and a basal position of *Nomascus* was indicated based on sequences of mtDNA control region and cytochrome b gene [10,20,23] but this result conflicts with the basal placement of the genus *Hoolock* in studies based on sequences of mtDNA ND3-ND4 genes [24], the concatenated sequences of mtDNA, Y-linked and X-linked loci [17], sequences of autosomal and X chromosomal regions [22] and chromosomal analysis [3], as well as with the basal position of the *Symphalangus* genus in an *Alu*-based phylogeny of gibbons [27]. Moreover, the evolutionary relationships within the two polyspecific genera, *Hylobates* and *Nomascus*, have been the particular focus of research using sequence data from the mtDNA and nuclear loci (e.g. [10,12,17-19,25,28,29]). MtDNA cytochrome b gene sequence analyses suggested that the divergences among *Nomascus* species accompanied a successive migration from north to south in which the two northernmost species *N. hainanus* and *N. nasutus* diverged first, followed by *N. concolor* and the two groups of southern species (the species *N. leucogenys* and *N. siki* and the two southernmost species *N. annamensis* and *N. gabriellae*) diverged last (Figure 1B; [10,12]). In contrast to the pattern found in the genus *Nomascus*, the biogeographic scenario for the dispersal of *Hylobates* species is still in question [10,17,20,25]. Nonetheless, the phylogenetic tree inferences from several datasets of mtDNA sequences (Figure 2B; [24,25]) and the concatenated sequences of the mtDNA, Y-linked and X-linked loci [17] suggest that the two northernmost mainland species (*H. lar* and *H. pileatus*) may have branched off earlier than other *Hylobates* gibbons, although the tree inferences from other sequence datasets of mtDNA cytochrome b gene and Y chromosome placed *H. klossii*[10], *H. moloch*[20] or *H. muelleri* (Figure 2C [19]) as basal species in *Hylobates* phylogeny Figure 2.

Although sequence data have thus been utilized to elucidate the phylogenetic relationships of *Hylobates* and *Nomascus* gibbons, most of these data were derived from single uniparentally inherited loci such as the maternally-inherited mtDNA [10,12,18,20,24,25,29] or the Y chromosome [28]. While mtDNA in particular provides useful information due to its rapid rate of evolution, DNA sequence data from multiple autosomal loci are needed to provide more comprehensive insights into the evolutionary relationships of species [30-32]. Recent advances in sequencing technologies have facilitated the gathering of multilocus sequence data from multiple individuals with extreme efficiency and thereby it has been feasible to acquire such data to explore evolutionary questions on species or population levels, even for non-model species [33,34]. A number of analytical approaches have been developed that process multilocus data to estimate parameters in a coalescent framework with use of the Bayesian Markov chain Monte Carlo (MCMC) method, which enables multilocus species tree inference in contrast to the trees inferred from single genes or concatenated sequences of multiple genes (e.g. [35-41]). In addition, multilocus data are suitable for the investigation of whether the divergence of closely related species was accompanied by gene flow (e.g. [39,40,42-51]).

These coalescent-based approaches provide an opportunity to use multilocus sequence data to re-examine the phylogenetic relationships of *Hylobates* species and *Nomascus* species through reconstruction of species trees as well as to investigate the role of gene flow in the evolutionary histories of these gibbons. Among the *Hylobates* species, three areas of sympatry with natural hybridization have been documented between species through the observations of wild populations: *H. agilis* and *H. lar* in the north-western part of peninsular Malaysia, *H. lar* and *H. pileatus* in eastern central Thailand and, *H. muelleri* and *H. albibarbis* in central Borneo, respectively [52-54]. Also, small areas of sympatry between *N. concolor* and *N. leucogenys* in regions of northern Vietnam, northwestern Laos and southern China have been reported and possible hybrid individuals between *N. concolor* and *N. leucogenys* have been identified [9,53,55]. A recent study of a large amount of nuclear sequence data (16 nuclear loci with a total length of ~40 kb) from a limited number of individuals detected a signal of gene flow between *N. gabriellae* and *N. leucogenys*[22]. These findings highlighted the possibility that gene flow may occur between other pairs of co-generic gibbon species during their divergences as well.

In this study, we generated multilocus autosomal DNA sequence data to investigate the evolutionary histories of gibbons. Specifically, we sequenced 14 autosomal noncoding loci, which in previous studies were useful for elucidating evolutionary histories of great apes, from each of 44 gibbon individuals. We examined the levels of genetic variation and differentiation among the gibbon taxa, and further compare levels of genetic variation and patterns of genetic diversity between the gibbon and great ape families. We reconstructed evolutionary relationships among the sampled gibbon taxa using coalescent species tree analysis employed in the program *BEAST and we further applied the isolation-with-migration (IM) model implemented in the program IMa2 to assess the possibility of gene flow between closely related species.

## Results and discussion

### Levels of genetic diversity in gibbons

We amplified and sequenced a total of ~11.5 kb from 14 autosomal noncoding loci from each of 44 gibbon individuals (Table 1). These individuals represent six *Hylobates*, four *Nomascus* and one *Symphalangus* species (Table 2). We observed a total of 713 polymorphic sites among all gibbons. The nucleotide diversity levels were generally similar among regions with relatively lower diversity levels in locus 4 and locus 10 (Table 1). The average values of π and θ_w_ across all loci were calculated for genera and species (Table 2). Of the three sampled genera, we observed the highest diversity level in *Hylobates* (N = 58, π = 0.61% and θ_w_ = 0.76%) and the lowest level in *Symphalangus* (N = 12, π = 0.17% and θ_w_ = 0.19%), consistent with results based on sequence data of mtgenomes and Y chromosomes (Table 2), although comparison to the genus *Hoolock* cannot be made since these data are lacking from this genus, which is not in captivity in Europe and so not readily sampled by us. However, comparative sequence data from 1140 bp of the mitochondrial cytochrome b gene and from 20 nuclear loci (17 autosomal and three X chromosomal loci, totaling ~60 kb in length), also suggested that *Hylobates* had highest genetic diversity and *Symphalangus* was the least diverse among the four existing gibbon genera (Table 2).

**Table 1:** Polymorphism and summary statistics of the sequenced loci

**Locus**	**Chromosome**^**a**^	**L (bp)**	**S**	**π (%)**	**θ**_**w **_**(%)**	**Dxy (%)**^**b**^
1	12q13.1	893	55	0.72	1.23	4.51
2	6p23	1056	77	1.01	1.46	3.63
3	5p15.2	836	45	0.92	1.08	3.58
4	20p12.3	985	44	0.67	0.89	3.05
5	5q23.3	872	56	0.99	1.28	1.98
7	19q12	887	53	1.10	1.12	4.29
8	16p12.3	688	45	1.19	1.30	2.83
10	4q24	771	36	0.52	0.94	2.58
12	17p12	757	56	1.51	1.47	3.97
13	5q12.1	802	55	1.31	1.44	3.40
15	14q23.2	783	53	1.06	1.34	4.03
16	9p23	798	49	1.11	1.22	3.40
20	10p11.21	664	47	1.43	1.43	4.71
21	17p12	709	42	0.86	1.18	4.60

L is the averaged sequence length; S is the number of polymorphic sites; π and θ_w_ are two standard diversity indices calculated across 44 gibbon individuals. ^a^The chromosomal location according to the human genome; ^b^Dxy is the average pairwise divergence per site [56] compared to chimpanzees [57].

**Table 2:** Diversity levels within gibbon and great ape genera and species

**Gibbons**						
Sequence	Genus or species	N	L (bp)	π (%)	θ_w_ (%)	Data source
14 autosomal loci	*Hylobates*	58	11501	0.61	0.76	Present study
	*Nomascus*	18	11501	0.36	0.42	
	*Symphalangus*	12	11501	0.17	0.19	
	*H. agilis*	12	11501	0.26	0.26	
	*H. klossii*	2	11501	0.08	0.08	
	*H. lar*	22	11501	0.35	0.30	
	*H. moloch*	8	11501	0.17	0.16	
	*H. muelleri*	6	11501	0.44	0.45	
	*H. pileatus*	8	11501	0.06	0.07	
	*N. concolor*	2	11501	0.09	0.09	
	*N. gabriellae*	4	11501	0.26	0.24	
	*N. leucogenys*	8	11501	0.28	0.28	
	*N. siki*	4	11501	0.19	0.19	
20 nuclear loci^a^ (17 autosomal and three X chromosomal loci)	*Hylobates*	16	64785	0.53	0.50	[22]
	*Nomascus*	18	90202	0.30	0.33	
	*Symphalangus*	2	40266	0.15	0.15	
	*Hoolock*	2	25053	0.19	0.19	
	*H. agilis*	6	32213	0.28	0.26	
	*H. lar*	2	36620	0.24	0.24	
	*H. moloch*	2	26187	0.31	0.31	
	*H. muelleri*	2	31706	0.47	0.47	
	*H. pileatus*	4	47677	0.26	0.26	
	*N. gabriellae*	4	79835	0.23	0.23	
	*N. leucogenys*	14	88531	0.26	0.27	
Mtgenome	*Hylobates*	29	15225	3.92	3.95	[18]^b^
	*Nomascus*	9	15225	1.61	1.76	
	*Symphalangus*	11	15225	0.49	0.66	
	*H. agilis*	4	15225	1.08	1.11	
	*H. lar*	15	15225	0.28	0.42	
	*H. moloch*	3	15225	0.48	0.48	
	*H. muelleri*	4	15225	1.68	1.47	
	*H. pileatus*	2	15225	0.07	0.07	
Cytochrome b	*Hoolock*	5	1140	5.39	5.31	[10]^c^
	*Hylobates*	39	1140	19.3	1.68	
	*Nomascus*	37	1140	4.13	3.87	
	*Symphalangus*	4	1140	1.14	1.10	
	*H. agilis*	8	1140	1.67	1.62	
	*H. albibarbis*	2	1140	1.05	1.05	
	*H. klossii*	5	1140	0.70	0.72	
	*H. lar*	11	1140	1.30	1.56	
	*H. moloch*	3	1140	0.94	0.94	
	*H. muelleri*	6	1140	2.39	2.61	
	*H. pileatus*	4	1140	0.59	0.59	
	*N. gabriellae*	9	1140	0.46	0.48	
	*N. leucogenys*	8	1140	0.46	0.44	
Y chromosome	*Hylobates*	19	6137	1.00	1.10	[19]
	*Nomascus*	4	6137	0.23	0.22	
	*Symphalangus*	3	6137	0.02	0.02	
Great apes						
Sequence	Genus or species	N	L (bp)	π (%)	θ_w_ (%)	Data source
Autosomal loci	*Homo*	90	16001	0.12	0.14	[57,58]
	*Gorilla*	34	14017	0.14	0.15	[59]
	*Pan*	78	21742	0.24	0.36	[57,60]^d^
	*Pongo*	32	16001	0.36	0.35	[57]

N is the number of chromosomes; L is the averaged sequence length; π and θ_w_ are two standard diversity indices. ^a^The loci were aligned to the human chromosomes 7, 8, 20, 22 and X. ^b^Mtgenome sequences excluding the control regions from the 49 gibbon individuals were used for the diversity index calculation; ^c^Cytochrome b gene sequences from 85 gibbons were used for the diversity index calculation; ^d^Autosomal sequences of 25 regions from nine bonobo and 30 chimpanzee individuals were used for the diversity index calculation.

We next compared the diversity levels among eight extant genera of the two ape families, Hominidae and Hylobatidae. Among great apes, sequence data from 16–25 autosomal loci, of which 14 were the same ones analyzed in this work on gibbons, found that orangutans (genus *Pongo*) had the highest diversity level (N = 32, π = 0.36% and θ_w_ = 0.35) as compared to the other three genera of great apes (Table 2) [57,59,60]. Interestingly, we observed diversity levels in the gibbon genera *Nomascus* (N = 18, π = 0.36% and θ_w_ = 0.42%) and *Hylobates* (N = 58, π = 0.61% and θ_w_ = 0.76%) as high, or even higher than that seen in orangutans. The higher diversity level of *Hylobates* than that of *Pongo* also was observed in the sequence data of the 20 nuclear loci (N = 16, π = 0.53% and θ_w_ = 0.50%, Table 2) [20]. Although there are just four genera in each of the two ape families, the high taxonomic diversity of 14–19 nominal gibbon species stands in contrast to the mere seven species of great apes [1,7-12].

Within *Hylobates*, we observed considerable variation in the diversity levels among six *Hylobates* species (ranging from 0.06%-0.44% for π and 0.07%-0.45 for θ_w_), and found within *H. muelleri* (N = 6, π = 0.44% and θ_w_ = 0.45%) a level over six times higher than that of *H. pileatus* (N = 8, π = 0.06% and θ_w_ = 0.07%). This finding of relatively higher diversity of *H. muelleri*, also seen from mtgenome sequences (Table 2), was also evident in a study of 20 nuclear loci employing eight individuals representing five *Hylobates* species (*H. agilis*, *H. moloch*, *H. muelleri* and *H. pileatus*) (Table 2) [22]. A recent study of the mtDNA cytochrome b gene, which used fairly comprehensive sampling of extant gibbon species including the recently identified *H. albibarbis*, provided estimates consistent with those based on mtgenome and nuclear datasets of relatively small sample sizes (Table 2) and supported the inference that *H. muelleri* may be the most genetically diverse *Hylobates* species [10]. Among the four sampled *Nomascus* species, we observed that *N. gabriellae* and *N. leucogenys* had similar diversity levels, which concurs with diversity estimates based on sequences of mtDNA cytochrome b and 20 nuclear loci (Table 2).

### Sequence divergence and genetic differentiation between gibbon taxa

We also investigated the patterns of inter-genus and interspecies sequence divergence and genetic differentiation between gibbons by calculating the average number of differences per site (π_b_) and pairwise F_ST_ statistics, respectively, between genera (Table 3). Using our sequence data from 14 autosomal loci, we observed levels of sequence divergence and genetic differentiation between our three sampled gibbon genera (mean π_b_ = 1.47% and F_ST_ = 0.72) similar to levels observed based on analysis of 20 nuclear loci (17 autosomal and three X chromosomal loci) for the same three genera (mean π_b_ = 1.41% and F_ST_ = 0.77) [22]. These two estimates were slightly increased when the *Hoolock* (the unsampled genus in this study) was included (mean π_b_ = 1.50% and F_ST_ = 0.80) [22]. We observed slightly lower sequence divergence between *Nomascus* and *Symphalangus* (π_b_ = 1.38%) as compared to between *Hylobates* and *Nomascus* (π_b_ = 1.46%) and between *Hylobates* and *Symphalangus* (π_b_ = 1.58%). These inter-genus sequence divergence estimates are similar to those observed between African apes of different genera (π_b_ = 1.12%-1.55%) but much lower than the levels between orangutans and any of the four African ape species (gorillas, bonobos, chimpanzees and humans; π_b_ = 3.02%-3.19%) [57]. Moreover, we also observed lower levels of genetic differentiation between the three gibbon genera (F_ST_ = 0.65-0.79) as compared to the levels between humans and orangutans (F_ST_ = 0.94), between human and gorillas (F_ST_ = 0.92) or between human and chimpanzees (F_ST_ = 0.89) [57]. In sum, these results are consistent with a relatively low level of genetic differentiation among the gibbon genera as compared to among the great ape genera.

**Table 3:** **Average values of pairwise π**_**b **_**and F**_**ST **_**between gibbon taxa**

**Among genera**						
Genus	*Hylobates*	*Nomascus*	*Symphalangus*			
*Hylobates*	-	0.65	0.73			
*Nomascus*	1.46	-	0.79			
*Symphalangus*	1.58	1.38	-			
Between *Hylobates*						
Species	*agilis*	*klossii*	*lar*	*moloch*	*muelleri*	*pileatus*
*H. agilis*	-	0.59	0.53	0.51	0.28	0.73
*H. klossii*	0.50	-	0.64	0.64	0.45	0.84
*H. lar*	0.66	0.62	-	0.59	0.36	0.76
*H. moloch*	0.53	0.60	0.67	-	0.33	0.77
*H. muelleri*	0.52	0.56	0.61	0.56	-	0.65
*H. pileatus*	0.88	0.86	0.92	0.88	0.91	-
Between *Nomascus*						
Species	*concolor*	*gabriellae*	*leucogenys*	*siki*		
*N. concolor*	-	0.56	0.30	0.46		
*N. gabriellae*	0.46	-	0.38	0.48		
*N. leucogenys*	0.30	0.46	-	0.34		
*N. siki*	0.31	0.47	0.35	-		

Below diagonal: π_b_ (%) values; above diagonal: F_ST_ values.

For the differentiation levels between species within the same genus, we found that within the genus *Hylobates*, the π_b_ values were higher in the species comparisons including *H. pileatus* (ranging from 0.86% to 0.92%) than those in other species pairs (ranging from 0.50% to 0.67%) (Table 3). Relatively higher F_ST_ values were consistently found in species pairs including *H. pileatus* (ranging from 0.65 to 0.84) compared to those of other species pairs (ranging from 0.28 to 0.64). The higher pairwise π_b_ and F_ST_ values estimated between *H. pileatus* and other *Hylobates* species were in general agreement with results obtained using data from 20 nuclear loci, in which five *Hylobates* species (*H. agilis*, *H. moloch*, *H. muelleri* and *H. pileatus*) were sampled and the highest π_b_ and F_ST_ values were observed between *H. muelleri* and *H. pileatus* (π_b_ = 0.82%) and between *H. agilis* and *H. pileatus* (F_ST_ = 0.66), respectively [22]. The relatively high levels of divergence and genetic differentiation between *H. pileatus* and other *Hylobates* species are consistent with phylogenetic analyses suggesting that *H. pileatus* diverged initially from the others during *Hylobates* evolution (Figure 2A, B; [17,24,25]). Moreover, although analyses using sequences of 20 nuclear loci found no evidence for genetic differentiation between *H. moloch* and *H. muelleri* (F_ST_ = 0.00) [22], our data from 14 autosomal loci showed a level of genetic differentiation between these two species (F_ST_ = 0.33) similar to the level between eastern and western chimpanzees (F_ST_ = 0.32) [57]. Within the genus *Nomascus*, the levels of divergence and genetic differentiation between our four sampled *Nomascus* species (π_b_ = 0.30%-0.46% and F_ST_ = 0.30-0.56) (Table 3) were similar to those between bonobos and three chimpanzee subspecies (π_b_ = 0.31%-0.32% and F_ST_ = 0.49-0.68) [57].

### Patterns of gibbon divergence

To elucidate the divergence processes in gibbon evolutionary histories, we analyzed our multi-locus sequence data using two coalescent-based approaches: the reconstruction of a species tree and the isolation-with-migration (IM) model for population/species divergence. We first inferred a species tree for the sampled gibbon species using the coalescent-based Bayesian MCMC method implemented in the program *BEAST [35,61,62] and the sequence data of 14 autosomal loci. We found that the monophyly of the three sampled gibbon genera previously suggested was well supported [3,10,17,19-21,24-26] (Figure 2). Within the genus *Hylobates*, our species tree of biparentally inherited multilocus data suggests that the species *H. pileatus* is the most basal taxon and the four species with geographic distribution restricted to the Sundaic inlands (*H. agilis*, *H. klossii*, *H. moloch* and *H. muelleri*; Figure 1A) cluster together (Bayesian posterior probability, PP =0.85) (Figure 2A). These results are largely consistent with those in the single locus mtDNA analyses (Figure 2B; [18,24,25]) as well in the tree generated using a concatenated dataset of mtDNA, Y-linked and X-linked loci [17]. However, our data here are insufficient to resolve the relationships among the four Sundaic species while the mtgenome tree showed closer relationships of *H. agilis*-*H. muelleri* and *H. klossii*-*H. moloch* (Figure 2B). Because of a higher mutation rate, a smaller effective population and consequently a shorter coalescence time than typical autosomal loci, the uniparentally inherited mtDNA is known to be conducive to resolving phylogenetic relationships of recently diverging taxa [31,32]. The divergence of the six *Hylobates* species analyzed here was estimated to occur over a short interval of about one million years [10,17] and thus an even shorter time for the divergence of four Sundaic species could be expected. Compared to the biparentally inherited autosomal loci, the mtgenome sequence data provided higher resolution for disentangling the phylogenetic relationships of the four Sundaic species.

**Figure 3:**
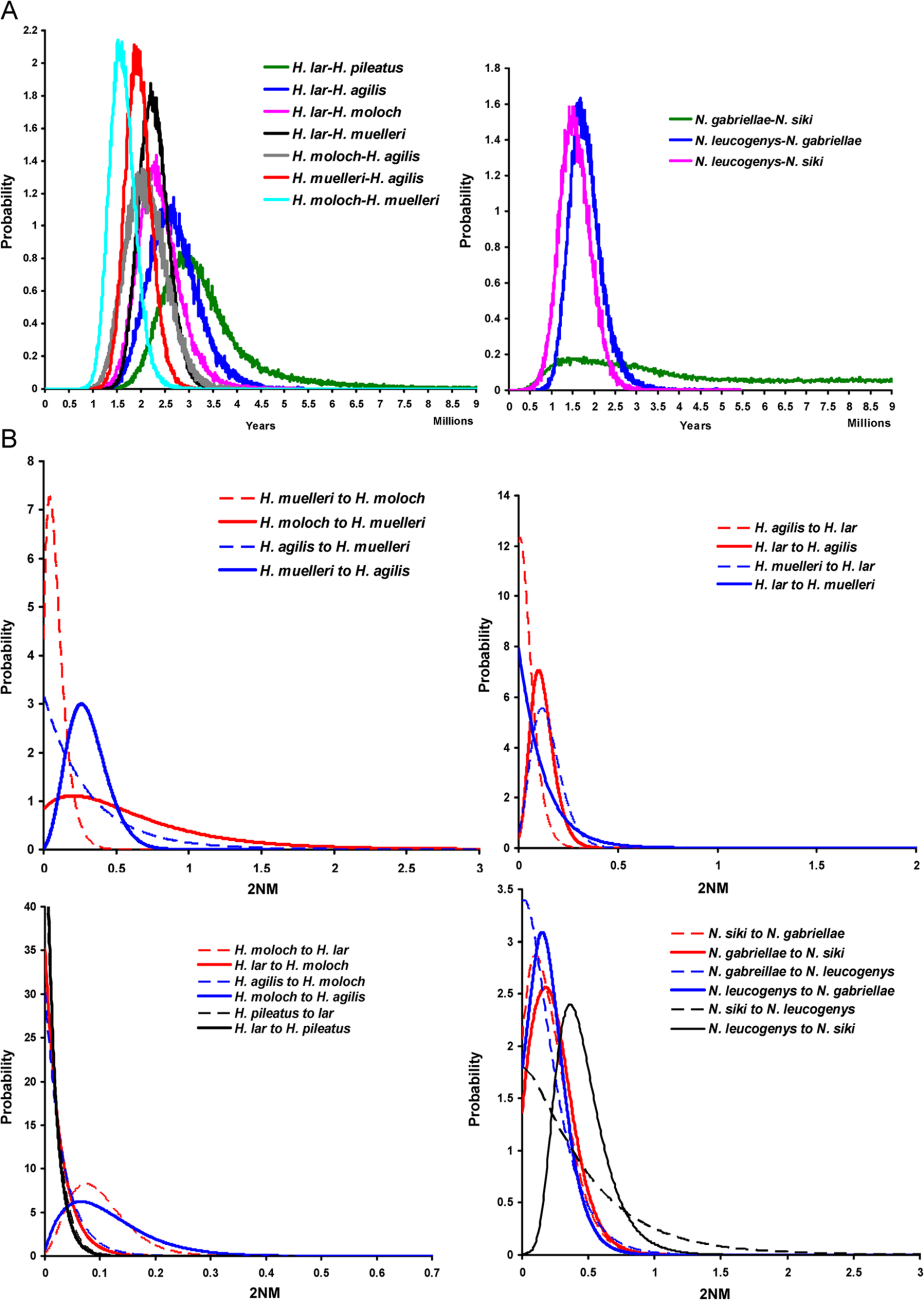
**Marginal posterior probability distribution for parameters in IMa2 pairwise comparison analyses.** Curves are shown for (**A**) estimates of divergence time parameter (in years) and (**B**) estimates of population migration rate (2NM) in each pairwise comparison analyses of *Hylobates* or *Nomascus* species.

We next used a program (IMa2) based on the isolation-with-migration model [49-51,63] to estimate multiple demographic parameters (including divergence time, migration rate and effective population sizes of derived populations and their ancestral populations) using MCMC simulation. Since the number of loci in our dataset (14) was insufficient for including all five sampled *Hylobates* species in a single IMa2 analysis, we conducted pairwise comparisons for these species as well as for the four *Nomascus* species studied. Species pairs were chosen for analysis based upon their close phylogenetic relationships or geographical proximity and consequent potential hybridization between them. Namely, IMa2 analyses were run for comparisons of seven *Hylobates* species pairs and for comparisons of three *Nomascus* species pairs (Table 4). The marginal posterior probability distributions of divergence time parameters showed clear peaks and bounds within the prior distribution for all pairwise comparisons except for *N. gabriellae* × *N. siki* which was tailed at the upper limit of the parameter prior space and hence returned an unreliable 95% highest posterior density (HPD) interval (Figure 3A). For *Hylobates* species, we found that the divergence time estimates were fairly consistent with the branching patterns of the species tree: (((*H. agilis*, *H. muelleri*, *H. moloch*, *H. klossii*), *H. lar*), *H. pileatus*) (Figures 2A and 3A). In the seven comparisons involving *Hylobates*, we found the oldest divergence time estimates between *H. lar* and *H. pileatus* (3 MYA) (Table 4 and Figure 2A). Following that, relatively younger time estimates were observed in pairwise comparisons between *H. lar* and the three species *H. agilis*, *H. moloch* and *H. muelleri* (2.2-2.5 MYA, *H. lar* × *H. agilis*, *H. lar* × *H. moloch* and *H. lar* × *H. muelleri*), which were older than the estimates of comparisons among the three species (1.5-2.1 MYA, *H. moloch* × *H. agilis*, *H. muelleri* × *H. agilis*, and *H. moloch* × *H. muelleri*) clustered together in the species tree (Table 4 and Figure 2A). Likewise, the younger estimate of divergence between *N. leucogenys* and *N. siki* (1.4 MYA) as compared to those between *N. leucogenys* and *N. gabriellae* and between *N. gabriellae* and *N. siki* (1.7-1.8 MYA) was consistent with branching patterns showing the prior divergence of *N. gabriellae* and the later divergence of the clade containing *N. leucogenys* and *N. siki* in the species tree (Table 4 and Figure 2A).

**Table 4:** IMa2 estimates and 95% highest probability density intervals (HPD) of demographic parameters

**Species 1 × species 2**	***m***_**1**_	***m***_**2**_	**t (years)**	**N**_**e1**_	**N**_**e2**_	**N**_**eA**_	**2N**_**1**_**M**_**1**_	**2N**_**2**_**M**_**2**_
***Hylobates *comparsions**								
*H.lar* × *H. agilis*								
Peak value	0.0035	0.1025*	2487549	63334	42819	39479	0.0079	0.1009*
Lower 95% HPD	0.0005	0.0125	1686037	49260	30415	8468	0.0004	0.0153
Upper 95% HPD	0.1055	0.2605	3758517	80748	59278	82894	0.1435	0.2417
*H. lar* × *H. moloch*								
Peak value	0.0528*	0.0003^a^	2315183	61664	32084	43296	0.0744*	0.0003^a^
Lower 95% HPD	0.0068	0.0003^a^	1557155	47590	21588	16817	0.0101	0.0003^a^
Upper 95% HPD	0.1522	0.1177	3319799	79555	47113	84326	0.2040	0.0836
*H. lar* × *H. muelleri*								
Peak value	0.0898*	0.0003^a^	2205588	55342	161665	34623	0.1175*	0.0021^a^
Lower 95% HPD	0.0013	0.0003^a^	1685015	40076	97326	16630	0.0090	0.0021^a^
Upper 95% HPD	0.2557	0.0873	2881421	73881	307791	61885	0.2914	0.3667
*H. lar* × *H. pileatus*								
Peak value	0.0003^a^	0.0003^a^	3000898	63214	11075	67645	0.0002^a^	0.0002^a^
Lower 95% HPD	0.0003^a^	0.0003^a^	1770005	49583	5623	13461	0.0002^a^	0.0002^a^
Upper 95% HPD	0.0411	0.2091	5624895	79913	18913	141253	0.0577	0.0508
*H. moloch* × *H. agilis*								
Peak value	0.0005^a^	0.0545*	2138864	30608	50112	22304	0.0004^a^	0.0650*
Lower 95% HPD	0.0005^a^	0.0015	1318541	23180	36015	96.55	0.0004^a^	0.0018
Upper 95% HPD	0.1455	0.2395	2968456	45863	69229	53008	0.0986	0.2593
*H. moloch* × *H. muelleri*								
Peak value	0.0516	0.0396	1520623	29171	215645	34623	0.0423	0.2063
Lower 95% HPD	0.0004	0.0004	1100511	17175	122408	17175	0.0006	0.0048
Upper 95% HPD	0.3740	0.2916	2169057	46073	423929	60795	0.2239	1.6740
*H. muelleri* × *H. agilis*								
Peak value	0.0006^a^	0.2586*	1867672	229719	37929	23616	0.0051^a^	0.2606*
Lower 95% HPD	0.0006^a^	0.0330	1429294	137163	22185	8349	0.0051^a^	0.0584
Upper 95% HPD	0.1710	0.8238	2506973	416738	58444	47948	1.0010	0.5714
***Nomascus *comparisons**								
*N. gabriellae* × *N. siki*								
Peak value	0.0730	0.1350	1768642	26922	21696	37599	0.1002	0.1790
Lower 95% HPD	0.0010	0.0010		13063	10110		0.0018	0.0020
Upper 95% HPD	0.9590	1.1910		49640	42597		0.5158	0.5411
*N. leucogenys* × *N. gabriellae*								
Peak value	0.0053	0.1477	1676632	97877	27950	22554	0.0153	0.1512
Lower 95% HPD	0.0008	0.0008	1093218	63345	13489	2698	0.0022	0.0017
Upper 95% HPD	0.2542	0.8363	2614455	155503	53417	48669	0.5773	0.4736
*N. leucogenys* × *N. siki*								
Peak value	0.0028^a^	1.1960*	1414914	102194	10468	15216	0.0061^a^	0.3641*
Lower 95% HPD	0.0028^a^	0.2447	864215	64856	3561	108	0.0061^a^	0.1268
Upper 95% HPD	0.5803	3.9410	2309117	166510	25575	37878	1.4320	0.8551

Missing values are where the HPD of the parameters could not be reliably estimated by IMa2; *m*_1_ is migration rate into species 1 from species 2; *m*_2_ is migration rate into species 2 from species 1; t is the time since the species 1 and 2 split in years; N_e1_, N_e2_ and N_eA_ are effective population sizes for species 1, species 2 and their ancestral population, respectively; 2N_1_M_1_ is population migration rate into species 1 from species 2 per generation; 2N_2_M_2_ is population migration rate into species 2 from species 1 per generation; ^a^The value corresponds to the first bin of the parameter space and hence could be interpreted as zero. *Asterisks indicates the estimates of migration rate that are significantly different from zero by the LLR tests [49,63] at the P < 0.05 level and bold text indicates the significance after Bonferroni correction at the P < 0.0035 level (for the *Hylobates* comparisons) or at the P < 0.008 level (for the *Nomascus* comparisons).

Our analyses suggest that *H. muelleri* has the largest and *H. pileatus* the smallest effective population sizes among the five analyzed *Hylobates* species (202,300 and 11,100, respectively) (Table 4). Given the relatively larger inferred effective population sizes of *H. muelleri* in all pairwise comparison analyses and the smaller population size of *H. pileatus* when compared to those of their respective ancestral population sizes (Table 4), we would suggest that the species *H. muelleri* has experienced population expansion while the *H. pileatus* population has decreased in size since the divergence. Similarly, *N. leucogenys* also appears to have expanded as the current effective population size is larger than those of ancestral populations in both comparisons *N. leucogenys* × *N. gabriellae* and *N. leucogenys* × *N. siki* (Table 4).

### Unidirectional gene flow between gibbon species

In addition to the estimations of divergence time and effective population sizes, the IMa2 analyses also provide inferences on the extent and patterns of gene flow in the divergence processes of species. Migration parameters (*m*) in the IM model can be transformed to obtain the estimates of population migration rate (2NM) which is the effective number of gene migrations received by a population per generation [49,64,65]. We found population migration rates significantly different from zero (2NM = 0.065-0.261, P < 0.05) in the comparisons of *H. lar* × *H. agilis*, *H. lar* × *H. moloch*, *H. lar* × *H. muelleri*, *H. moloch* × *H. agilis*, and *H. muelleri* × *H. agilis* (Table 4 and Figure 3B). However, after applying Bonferroni correction for multiple testing, significantly nonzero population migration rates were only found in the comparisons *H. lar* × *H. moloch* and *H. muelleri* × *H. agilis*, which indicated gene flow from *H. moloch* to *H. lar* (2NM = 0.074, P < 0.0035) and from *H. muelleri* to *H. agilis* (2NM = 0.261, P < 0.0035) (Table 4). We also detected significantly nonzero population migration rate for the gene flow from *N. leucogenys* to *N. siki* (2NM = 0.364, P < 0.008) (Table 4). Also notable is the asymmetry in gene flow, as we only found significant signals of gene flow in one direction but not in opposite direction in the comparisons where significantly nonzero migration rates were observed (Table 4 and Figure 3B).

Among the *Hylobates*, most species are currently separated from each other by bodies of water (e.g. the Java Sea and Karimata Strait between *H. lar* and *H. moloch* and the Karimata Strait between *H. agilis* and *H. muelleri*). Given these barriers, it is necessary that any signal of gene flow found between such species cannot be the consequence of recent contact. Rather, lower sea levels and changes in the distributions of gibbon populations in the past may have allowed some interactions among these *Hylobates* taxa [10,66,67]. Furthermore, it is possible that the signals of gene flow we detected between *Hylobates* species may underestimate the history of gene flow during *Hylobates* divergence. Our dataset was insufficient for attempting IMa2 analyses in the multi-population model which can reveal historical gene flow involving ancestral populations [49,64]. Consequently, we could only assess gene flow between two derived populations and any gene flow between the derived populations and the ancestral populations was not addressed in our pairwise comparison analyses. The importance of this consideration was shown in an analysis of bonobos and three chimpanzee subspecies in which asymmetric gene flow was detected in a pairwise analysis between allopatric central and western chimpanzees while further three- and four-population analyses suggested that there had actually been gene flow between the western chimpanzees and the ancestral population of central and eastern chimpanzees [64]. Accordingly, that the gene flow from the western to the central chimpanzees identified in the two-population analysis likely reflects the history of gene flow from the western chimpanzees into the ancestor of central and eastern chimpanzees identified in multi-population analyses [64]. In the case of *Hylobates* species, the four Sundaic species (*H. agilis*, *H. klossii*, *H. moloch* and *H. muelleri*) shared the same ancestral population according to phylogenetic analyses (Figure 2A, B; [17,24,25]) and the gene flow suggested by our IMa2 analyses of the comparisons among *H. lar* and three of these species (*H. agilis*, *H. moloch* and *H. muelleri*) could reflect historical gene flow between *H. lar* and the ancestral population of the Sundaic species. Our analyses did not allow us to date the time of potential gene flow between species. However, mtDNA studies have supported the monophylies of *Hylobates* species, while Y-chromosome studies are less clear on the possibility of sharing of Y-haplotypes between groups (Figure 2B, C; [10,19,24,25]). Thus, it is possible that any female-mediated gene flow in *Hylobates* may have occurred deep enough in the past to allow for mitochondrial lineage sorting, or that any more recent gene flow has been male-mediated.

Among *Nomascus* species, *N. leucogenys* and *N. siki*, who share more similarities of genetics, morphology and acoustics to each other than to other *Nomasacus* species [12,53,68], exist in adjacent distribution areas (Figure 1B), which might allow some gene exchanges between these two species in their contact zones. We detected a significant signal of gene flow between *N. leucogenys* and *N. siki* (Table 4 and Figure 3B), but cannot assess whether this may be a result of gene exchanges occurring during their divergence processes or a consequence of relatively recent secondary contact after speciation. In the case of secondary contact after speciation, the exchanged alleles may have not spread over the ranges of the two species. Analyses with geographically selective sampling, where individuals would be sampled far from and in/near the contact zones, may be helpful to distinguish between the scenarios of divergence with gene flow and secondary contact, as the signal of gene flow could be reduced or eliminated when excluding particular individuals from or near contact zones (e.g. [40,45]). Our sampling of *N. leucogenys* and *N. siki* is limited and the provenances of these individuals are unclear. The contemporary geographic distributions of *Nomascus* species have been recently revised via vocal and genetic analyses of individuals with known geographic origins and especially those from areas of potential species boundaries, but because these noninvasively collected samples and tissue samples from museum specimens yield DNA of poor quality, limiting the scope of the genetic analyses to the *Nomascus* phylogeny based on mtDNA cytochrome gene sequence data [12,68].

Gene flow between *N. leucogenys* and *N. gabriellae* with a migration rate equivalent to a rate about one migrant every two generations has also been suggested [22]. However, although we obtained population migration rate estimates of 2NM = 0.015 for migration from *N. gabriellae* into *N. leucogenys* and 2NM = 0.151 from *N. leucogenys* into *N. gabriellae*, these two estimates were not significantly different from zero (P > 0.1), indicating no gene flow between these two species whose geographic distributions are currently discontiguous and interrupted by the distributions of *N. siki* and *N. annamensis* (Table 4 and Figure 1B).

The detection of asymmetric gene flow between gibbon species is not unexpected but has also been seen for closely related taxa in other primates (e.g. gorillas [59], macaques [45], chimpanzees [64,69], and baboons [70]) and other animals (review in [65]). For example, more gene flow may take place from eastern to western gorillas (2NM = 0.350) than western to eastern gorillas (2NM = 0.141) [59]. Unidirectional patterns of gene flow apparently occurred between three *Mus* species [43] as well as between two macaque species [45]. In the case of macaques, the IM analyses with exclusion of loci violating neutrality found that the gene flow from rhesus into cynomologus macaques was estimated as 2NM = 0.493, while gene flow in the other direction was not significantly different from zero [45]. Moreover, the extent of gene flow we detected here between gibbons species was similar to that estimated between other closely related taxa (e.g. gorillas [59] or *Mus* species [43]). However, the population genetic structure within species might lead to different inferences about the extent of gene flow between species. For example, a signal of gene flow may be underestimated or not be detected if samples are not collected from populations in potential contact zones [45]. The magnitude of gene flow detected between two species would also increase with decreasing distance between the sampled populations of two species [70]. Our sample sizes of each gibbon species are limited and their geographic origins are unclear. Therefore, the extent and patterns of gene flow detected here likely represent a minimum estimate and additional work is needed incorporating extensive sampling of individuals with known geographic provenance.

## Conclusions

Our analyses of sequence data of 14 autosomal loci, coupled with two coalescent-based analyses (*BEAST and IMa2), provide inferences of species trees and the extent and patterns of gene flow among gibbon taxa. Our tree (Figure 2A), like those based upon mtDNA sequences or the concatenated sequences of mtDNA, Y-linked and X-linked loci, shows *H. pileatus* as the basal *Hylobates* taxon and groups the four Sundaic species (Figure 2B; [17,24,25]). We find evidence for unidirectional gene flow between some gibbon species; namely between *H. lar* and *H. moloch*, between *H. agilis* and *H. muelleri* and between *N. leucogenys* and *N. siki*. Further insights will require the use of multi-population analyses investigating historical gene flow involving ancestral populations [49,64] by use of a larger dataset of more loci as well as a larger set of samples of known geographic origin.

## Methods

### Gibbon DNA samples and PCR amplification of 14 autosomal loci

We used 44 high-quality genomic DNA samples, including representatives of six *Hylobates*, four *Nomascus* and one *Symphalangus* species. All DNA samples used derive from the long-term sample collections of the authors and were not acquired specifically for this study. These samples were originally collected in the course of routine veterinary examinations of captive gibbons. We performed whole genome amplification (WGA) on all genomic DNA samples using the multiple displacement amplification procedure implemented in the GenomiPhi HY DNA Amplification Kit (GE Healthcare). The WGA products were purified by ethanol precipitation following manufacturer’s instructions. The purified WGA products were quantified using a NanoDrop spectrophotometer (Thermo Fisher Scientific, Inc.) and used as templates for subsequent polymerase chain reactions (PCRs) for the amplification of autosomal loci.

We amplified 14 autosomal loci (Table 1) previously shown to be noncoding and selectively neutral and used in studies on the evolutionary histories of great apes [57-60,71]. The polymerase chain reaction (PCR) primers used for these 14 loci were described in the previous studies [57,59], which were designed by using the human and chimpanzees. The PCR amplification reactions were carried out in a volume of 50 μl containing 60–100 ng of purified WGA products, 0.4 mM MgCl_2_, 0.2 μM of each forward and reverse primer, 200 μM of each dNTP, 10 × SUPER TAQ PCR buffer (containing MgCl_2_), and 1.5 units of SUPER TAQ (HT Biotechnology, Cambridge, UK) premixed 2:1 with 1 μg/μl TaqStart monoclonal antibody (BD Bioscience Clontech). The PCR condition included following steps: initial denaturation at 94°C for 1 min; 35 cycles of denaturation at 94°C, primer annealing at 57–61°C for 1 min 30 sec, elongation at 72°C, and a final elongation step of 7 min at 72°C. The PCR products were gel-cut and purified using QIAquick Gel Extraction Kit (Qiagen).

### Sequencing of 14 autosomal locus amplicons

We used the high-throughput Illumina sequencing platform to sequence 14 autosomal loci for 44 gibbons. A sequencing library containing 14 autosomal locus amplicons was created for each of 44 individuals using a modified Illumina protocol [72] where a PCR reaction was used to add individual-specific indexing oligos to both ends of library molecules. This indexing PCR procedure allowed us to identify and sort read sequences by individuals during data processing. The 44 individual indexed libraries were pooled in equimolar ratio and sequenced on a single lane of the flow cell of an Illumina Genome Analyzer IIx instrument with paired-end sequencing of 76 + 7 cycles according to the manufacturer’s instructions (Illumina). Bases and quality scores were generated with the Ibis base caller [73]. The reads were then processed based on their indexes and the indexed reads were aligned with the Burrows-Wheeler Aligner (BWA) software [74] to the chimpanzee homologues of 14 autosomal locus sequences with default parameters, resulting in the 44 individual BAM files (44 gibbons). Subsequently, the BAM files were processed separately and the reads of potential PCR duplicates were removed using SAMtools [75]. The consensus sequences of 14 autosomal loci of 44 gibbons were generated also by using SAMtools. In sum, for each consensus sequence, each base had a minimum averaged PHRED score of 25 and the alternate alleles of heterozygous sites had a coverage rate of at least 30%. The summary statistics of the reads for each gibbon is provided in Additional file 2 and the consensus sequences have been deposited in Genbank under the accession numbers KC480606-KC481221.

### Sequence data analysis

Multiple alignments for the consensus sequences were generated with ClustalW v2.0 [76] and then edited and checked with BioEdit v7.0.5 [77]. Haplotype phases were inferred with PHASE v2.1 [78,79] for each locus where the program SeqPHASE [80] was used to interconvert FASTA files of the alignments to the formats of PHASE input and output files. The program DnaSP v5.10 [81] was used to calculate two standard diversity indices, π [56] and θ_w_[82], and pairwise F_ST_ statistics between genera or species [83]. We also estimated the average number of differences per site between sequences sampled from two different genera or two species (nucleotide diversity between populations, π_b_). To test the selective neutrality of each locus, we calculated Tajima’s D [84] using DnaSP with 10,000 coalescent simulations, suggesting no signal of a departure from neutral evolution.

### Estimation of gene flow

We used program IMa2 [49] to assess the extents of gene flow that may have occurred between *Hylobates* species and between *Nomascus* species. Since IMa2 assumes no recombination in each locus analyzed, we tested the possibility of intralocus recombination with methods implemented in the programs Recombination Detection Program (RDP) v3.44 [85]: RDP [86], GENECONV [87], MaxChi [88], Chimaera [89], SiScan [90] and 3Seq [91]. When recombination was detected, only non-recombining blocks of sequences were used in the input datasets. The base positions with gaps/indels in the alignments were removed from IMa2 analyses. The program IMa2 is based on an isolation-with-migration (IM) model and estimates the posterior probability densities using Markov chain Monte Carlo (MCMC) simulation for the parameters scaled by the mutation rate (μ): bi-directional migration rate (*m* = M/μ, where M is the migration rate per generation per gene copy), population size (θ = 4N_e_μ, where N_e_ is effective population size) and divergence time (*t* = tμ, where t is the time since population splitting) [49-51,63]. The parameter estimates of population size and divergence time resulting from the IMa2 analyses were converted to the estimates of effective population size (N_e_) in individuals and divergence time (t) in years. The migration rate parameters can be transformed into the estimates of population migration rate (i.e. 2NM = 4N_e_μ × M/μ/2), the effective number of migrant gene copies per generation [49,64]. For these conversions, we used a time of splitting between gibbon and great apes of approximately 19.5 million years [15,92-94] and an assumed generation time of 15 years. The life histories of gibbons have been suggested to resemble those of great apes rather than same-sized monkeys [95]. The female age of first reproduction has been estimated for wild populations of *H. lar* at 11.06 years, which was only slightly younger than that of great ape females, and its interbirth interval has been estimated as 41 months, longer than those of same-sized monkeys (e.g. *Macaca* or *Cercocebus*) [95]. Therefore, we assumed a generation time of 15 years for gibbons. We estimated the mutation rate per year for each locus using the average divergence of sequences (D_xy_) [56] between gibbon and chimpanzee (Table 1) with the divergence time (T) of 19.5 million years between them (Dxy = 2Tμ approximately under a neutral evolution model). The average mutation rate (mutation/site/year) of the 14 loci was calculated as 0.94 × 10^-9^, which is similar to the commonly-used per-site genome-wide mutation rates of human and other great apes [14,96-98].

Due to the limited number of loci and large number of gibbon species, we were unable to include all *Hylobates* species in a single IMa2 analysis and thus we conducted the IMa2 analyses with pairwise comparisons between *Hylobates* species as well as between *Nomascus* species. Species for which we had sequenced only one individual were excluded from the analyses (i.e. *H. klossii* and *N. concolor*). The species pairs were selected for analysis based on: (1) evidence for close phylogenetic relationships or (2) the presence of contact zones or suggestions of potential hybridization. Accordingly, we conducted seven comparisons for the *Hylobates* spp. and three comparisons for the *Nomascus* spp. (Table 4). Preliminary runs were performed to estimate the settings of uniform priors (upper bound on the uniform prior distribution) for the parameters, the necessary duration of runs and the heating terms of Metropolis-coupled chains required for well-mixed Markov chains. Once optimal priors and heating schemes were devised from initial runs, 20 independent Markov chains (−hn20 -ha0.96 -hb0.9) and six independent runs with different starting seeds (adjusting only the starting seed) were performed for each analysis. We saved 20,000 genealogies per run after a sufficient burn-in period (1,000,000 burn-in steps). Stationarity was reached already during the burn-in period. The adequate convergence of the MCMC simulation was assessed by (i) inspection of autocorrelation values over the course the run and effective sample sizes (ESS); (ii) inspection of the parameter trend plots; and (iii) checking that the parameter estimates calculated using genealogies sampled in the first and second halves of the run were highly similar. At least 100,000 sampled genealogies pooled from independent MCMC runs were used to calculate marginal posterior probability density estimates for the parameters using “L-mode” in IMa2, and LLR test (likelihood ratio test) statistics for assessing whether the estimated migration rates are significantly different from zero with a mix chi-squared distribution [49,63].

### Bayesian inference of species tree

We conducted a coalescent-based method for species tree reconstruction employed in the program *BEAST [35] using chimpanzee sequences as the outgroup. This method jointly estimates the posterior distributions of species tree and a set of gene trees from multi-locus sequence data and is implemented in the BEAST software package v1.7.2 [61,62]. The best-fit substitution models were assessed using the Akaike information criterion (AIC) by Model-Generator v0.85 [99] and were set independently for each locus partition. In addition, the clock and tree models were unlinked for all locus partitions. Four independent BEAST runs of 100,000,000 generations (100 million) were carried out with lognormal relaxed clock model and Yule speciation process in tree prior, sampling every 5,000 generations. Convergence was assessed in Tracer 1.5 [100] and the burn-in period was set as 4,000 trees. We combined the log output files from four individual BEAST runs using LogCombiner 1.7.2 [62] and the ESS values for all parameters were above 200. The maximum-clade-credibility tree was generated using TreeAnnotator 1.7.2 [62] and visualized using FigTree 1.3.1 [101].

## Abbreviations

IM: Isolation with migration; mtDNA: Mitochondrial DNA; MCMC: Markov chain Monte Carlo; mtgenome: Mitochondrial genome; HPD: Highest posterior density; MYA: Million years ago; WGA: Whole genome amplification; PCR: Polymerase chain reaction; BWA: Burrows-Wheeler Aligner; ESS: Effective sample sizes; AIC: Akaike information criterion.

## Competing interests

The authors declare that they have no competing interests.

## Authors’ contributions

Y-CC carried out the experimental work of the study, analyzed the data and wrote the manuscript. CR and EI contributed samples and helped to draft the manuscript, and MI-M, KJ-CP and C-CS contributed samples. LV conceived of the study and wrote the manuscript. All authors read and approved the final manuscript.

## Supplementary Material

Additional file 1 **Reconstruction of the mtgenome phylogeny tree.** Bayesian analysis of gibbon phylogenetic relationships based on the mtgenome sequences, excluding the control regions, from 49 individuals. Click here for file

Additional file 2: Tables S1 to S2 This file includes details regarding gibbon samples used in the present study and summary statistics of the reads for each gibbon. Click here for file
