# Supplementary material for: Inferring the evolutionary histories of divergences in Hylobates and Nomascus gibbons through multilocus sequence data
Source: BMC Evol Biol. 2013 Apr 12;13:82. doi: 10.1186/1471-2148-13-82 (PMC3637282; doi:10.1186/1471-2148-13-82)
Supplement: Additional file 1 — Reconstruction of the mtgenome phylogeny tree. Bayesian analysis of gibbon phylogenetic relationships based on the mtgenome sequences, excluding the control regions, from 49 individuals. [file 1471-2148-13-82-S1.doc]

**Additional file 1: Reconstruction of the mtgenome phylogeny tree**

We retrieved and re-examined a total 51 of the mtgenome sequences which were previously deposited in the GenBank by Chan et al. . Of the 51 mtgenome sequences, we found that two sequences (accession numbers HQ622760 and HQ622786) were partially incorrect and therefore we removed these two sequences from our phylogenetic analyses for the reconstruction of the mtgenome tree. We re-analyzed 49 mtgenome sequences, excluding the control regions. The sequences were partitioned the sequences into three schemes comprising rRNA, tRNA and protein. The best-fit nucleotide substitution model was selected by Model-Generator 0.85 and the general time reversible (GTR) + I + Γ model was suggested for each partition. We conducted the partitioned Bayesian analysis implemented in the program MrBayes 3.1.2 . Four Metropolis-coupled Markov chain Monte Carlo (MCMC) analyses were run twice for 5,000,000 generations and sampled every 100 generations (mcmcp ngen=5000000, nchains=4, temp=0.01, samplefreq=100, burnin=5000). The GenBank IDs of the mtgenome sequences used for the mtgenome tree reconstruction are HQ622758-HQ622759, HQ622761- HQ622785, and HQ622787-HQ622808.

References:

1. Chan YC, Roos C, Inoue-Murayama M, Inoue E, Shih CC, Pei KJ, Vigilant L: **Mitochondrial genome sequences effectively reveal the phylogeny of *Hylobates* gibbons.** *PLoS One* 2010, **5:**e14419.

2. Keane TM, Creevey CJ, Pentony MM, Naughton TJ, McLnerney JO: **Assessment of methods for amino acid matrix selection and their use on empirical data shows that ad hoc assumptions for choice of matrix are not justified.** *BMC Evol Biol* 2006, **6:**29.

3. Ronquist F, Huelsenbeck JP: **MrBayes 3: Bayesian phylogenetic inference under mixed models.** *Bioinformatics* 2003, **19:**1572-1574.
