# Supplementary material for: Inferring the evolutionary histories of divergences in Hylobates and Nomascus gibbons through multilocus sequence data
Source: BMC Evol Biol. 2013 Apr 12;13:82. doi: 10.1186/1471-2148-13-82 (PMC3637282; doi:10.1186/1471-2148-13-82)
Supplement: Additional file 2: Tables S1 to S2 — This file includes details regarding gibbon samples used in the present study and summary statistics of the reads for each gibbon. [file 1471-2148-13-82-S2.doc]

**Additional file 2: Tables S1 to S2.**

Table S1. Gibbon samples used in the present study

| Genus | Species | owner's ID/Barcode ID | working ID | origin^a^ | current deposition^a^ |
| --- | --- | --- | --- | --- | --- |
| *Hylobates* | *agilis* | 20050292D10 | T01 | Taipei Zoo | Taipei Zoo |
|  |  | 20060466D10 | T02 | Taipei Zoo | Taipei Zoo |
|  |  | 1135 | 1135 | Bristol Zoo | German Primate Center |
|  |  | 1136 | 1136 | Bristol Zoo | German Primate Center |
|  |  | 1138 | 1138 | Bristol Zoo | German Primate Center |
|  |  | 1061 | J24 | Ragunan Zoo | WRC, Kyoto University |
|  | *lar* | 20040082D10 | T04 | Taipei Zoo | Taipei Zoo |
|  |  | 20040113D10 | T10 | Taipei Zoo | Taipei Zoo |
|  |  | 20040284D10 | T11 | Taipei Zoo | Taipei Zoo |
|  |  | 20040286D10 | T13 | Taipei Zoo | Taipei Zoo |
|  |  | 960530G01 | P01 | Pingtung Rescue Center | Pingtung Rescue Center |
|  |  | 23 | 23 | Wuppertal Zoo | German Primate Center |
|  |  | 26 | 26 | Wuppertal Zoo | German Primate Center |
|  |  | 501 | 501 | Nuremberg Zoo | German Primate Center |
|  |  | 502 | 502 | Nuremberg Zoo | German Primate Center |
|  |  | 1982 | J27 | Chiang Mai Zoo | WRC, Kyoto University |
|  |  | 3400 | J29 | PRI, Kyoto University | WRC, Kyoto University |
|  | *muelleri* | 20050386D10 | T16 | Taipei Zoo | Taipei Zoo |
|  |  | 2521 | J15 | Kalimantan Samarinda | WRC, Kyoto University |
|  |  | 1058 | 1058 | Rostock Zoo | German Primate Center |
|  | *klossii* | 1230 | 1230 | Twycross Zoo | German Primate Center |
|  | *moloch* | 2486 | J12 | Ragunan Zoo | WRC, Kyoto University |
|  |  | 2488 | J13 | Ragunan Zoo | WRC, Kyoto University |
|  |  | 2349 | J21 | Ragunan Zoo | WRC, Kyoto University |
|  |  | 1228 | 1228 | Twycross Zoo | German Primate Center |
|  | *pileatus* | 2360 | J16 | Dusit Zoo | WRC, Kyoto University |
|  |  | 2361 | J17 | Dusit Zoo | WRC, Kyoto University |
|  |  | 2362 | J22 | Dusit Zoo | WRC, Kyoto University |
|  |  | 1227 | 1227 | Twycross Zoo | German Primate Center |
| *Symphalangus* | *syndactylus* | 20040396D10 | T20 | Taipei Zoo | Taipei Zoo |
|  |  | 20060592D10 | T21 | Taipei Zoo | Taipei Zoo |
|  |  | 659 | 659 | La Vallee des Singes | German Primate Center |
|  |  | 980 | 980 | Krefeld Zoo | German Primate Center |
|  |  | 2507 | J20 | Ragunan Zoo | WRC, Kyoto University |
|  |  | 1973 | J33 | Padang, Sumatra, Indonesia | WRC, Kyoto University |
| *Nomascus* | *leucogenys* | 378 | 378 | Twycross Zoo | German Primate Center |
|  |  | 1005 | 1005 | Duisburg Zoo | German Primate Center |
|  |  | 1006 | 1006 | Duisburg Zoo | German Primate Center |
|  |  | 2364 | J04 | Dusit Zoo | WRC, Kyoto University |
|  | *siki* | 1232 | 1232 | London Zoo | German Primate Center |
|  |  | 1986 | J03 | Chiangmai Zoo | WRC, Kyoto University |
|  | *gabriellae* | Arnold | Arnold | Leipzig Zoo | MPI-EVA |
|  |  | Falco | Falco | Leipzig Zoo | MPI-EVA |
|  | *concolor* | 1231 | Nc | Twycross Zoo | German Primate Center |

^a^ Abbreviations: PRI, Primate Research Institute; WRC, Wildlife Research Center; MPI-EVA, Max-Planck Institute for Evolutionary Anthropology

Table S2. Summary statistics of the reads for each gibbon.

| working ID | Number of filtered paired-end reads | Number of mapped reads | Total resulted bases (Mb) | Average coverage |
| --- | --- | --- | --- | --- |
| 23 | 1092136 | 636828 | 47.17 | 4141 |
| 26 | 1217420 | 754083 | 55.89 | 4878 |
| 378 | 1323045 | 795094 | 59.06 | 5178 |
| 501 | 1536378 | 919640 | 68.27 | 5961 |
| 502 | 1286570 | 756437 | 56.10 | 4925 |
| 659 | 942210 | 565713 | 41.92 | 3701 |
| 980 | 1225788 | 742943 | 55.13 | 4862 |
| 1005 | 1462043 | 863923 | 64.21 | 5668 |
| 1006 | 1338386 | 835069 | 61.99 | 5479 |
| 1058 | 1392470 | 802631 | 59.49 | 5214 |
| 1135 | 1432791 | 816556 | 60.50 | 5299 |
| 1136 | 1312166 | 759734 | 56.30 | 4946 |
| 1138 | 1286848 | 755289 | 56.04 | 4930 |
| 1227 | 1480352 | 792920 | 58.77 | 5156 |
| 1228 | 1194690 | 693238 | 51.45 | 4491 |
| 1230 | 1420755 | 780805 | 57.92 | 5084 |
| 1232 | 835513 | 529079 | 39.22 | 3446 |
| Arnold | 1606344 | 958250 | 71.23 | 6247 |
| Falco | 1359397 | 798422 | 59.27 | 5212 |
| J03 | 1129486 | 704234 | 52.26 | 4589 |
| J04 | 1325928 | 797079 | 59.19 | 5233 |
| J12 | 1344070 | 757863 | 56.12 | 4939 |
| J13 | 1281049 | 723362 | 53.59 | 4718 |
| J15 | 1336017 | 805581 | 59.73 | 5231 |
| J16 | 1186467 | 719292 | 53.40 | 4665 |
| J17 | 1270795 | 758110 | 56.27 | 4898 |
| J20 | 1495204 | 874611 | 64.92 | 5719 |
| J21 | 1251137 | 748377 | 55.47 | 4865 |
| J22 | 1193266 | 727181 | 53.98 | 4707 |
| J24 | 1403975 | 839165 | 62.13 | 5455 |
| J27 | 1200468 | 737495 | 54.72 | 4785 |
| J29 | 1384612 | 841625 | 62.50 | 5454 |
| J33 | 1417897 | 856225 | 63.59 | 5623 |
| Nc | 1491835 | 794906 | 59.04 | 5190 |
| P01 | 1366697 | 659793 | 48.90 | 4326 |
| T01 | 1399517 | 810695 | 60.17 | 5303 |
| T03 | 1297500 | 749802 | 55.57 | 4860 |
| T04 | 1101171 | 670260 | 49.68 | 4342 |
| T10 | 1088009 | 632642 | 46.88 | 4126 |
| T11 | 1122739 | 685079 | 50.77 | 4438 |
| T13 | 1171624 | 713458 | 52.94 | 4619 |
| T16 | 1607196 | 760356 | 56.47 | 4981 |
| T20 | 1058540 | 636968 | 47.20 | 4207 |
| T21 | 1159544 | 724802 | 53.80 | 4751 |
| Mean | 1291592 | 756491 | 56.12 | 4928 |
